# Exploration of prognostic factors for critical COVID-19 patients using a nomogram model

**DOI:** 10.1038/s41598-021-87373-x

**Published:** 2021-04-14

**Authors:** Juan Li, Lili Wang, Chun Liu, Zhengquan Wang, Yi Lin, Xiaoqi Dong, Rui Fan

**Affiliations:** 1 Nursing Department, Ningbo Medical Center Lihuili Hospital, Ningbo, Zhejiang China; 2 Division of Cardiothoracic and Vascular Surgery Department, Tongji Hospital, Tongji Medical College, Huazhong University of Science and Technology, Wuhan, Hubei China; 3 Emergency Medicine Department, Yuyao People’s Hospital, Medical School of Ningbo University, Ningbo, Zhejiang China; 4 Department of Respiratory, The Affiliated Xiangshan Hospital of Wenzhou Medial University, Ningbo, Zhejiang China; 5 Respiration Department, Ningbo Medical Center Lihuili Hospital, Ningbo, Zhejiang China; 6 Medical Quality Management Office, Ningbo Medical Center Lihuili Hospital, Ningbo, Zhejiang China

**Keywords:** Diseases, Risk factors

## Abstract

The study aimed to explore the influencing factors on critical coronavirus disease 2019 (COVID-19) patients’ prognosis and to construct a nomogram model to predict the mortality risk. We retrospectively analyzed the demographic data and corresponding laboratory biomarkers of 102 critical COVID-19 patients with a residence time ≥ 24 h and divided patients into survival and death groups according to their prognosis. Multiple logistic regression analysis was performed to assess risk factors for critical COVID-19 patients and a nomogram was constructed based on the screened risk factors. Logistic regression analysis showed that advanced age, high peripheral white blood cell count (WBC), low lymphocyte count (L), low platelet count (PLT), and high-sensitivity C-reactive protein (hs-CRP) were associated with critical COVID-19 patients mortality risk (*p* < 0.05) and these were integrated into the nomogram model. Nomogram analysis showed that the total factor score ranged from 179 to 270 while the corresponding mortality risk ranged from 0.05 to 0.95. Findings from this study suggest advanced age, high WBC, high hs-CRP, low L, and low PLT are risk factors for death in critical COVID-19 patients. The Nomogram model is helpful for timely intervention to reduce mortality in critical COVID-19 patients.

## Introduction

The 2019 novel coronavirus, 2019-nCoV, also known as SARS-CoV-2, can cause serious pneumonia. Since its emergence, coronavirus disease 2019 (COVID-19) has spread across China and globally, with high morbidity and mortality^1^. As of 18:00 on April 27th, 2020 CEST (Central European Summer Time), there have been 2,810,325 global confirmed cases and 193,825 fatalities (WHO). The 6.9% death rate of COVID-19 has seriously influenced the general public’s safety^2,3^. Wuhan, China’s most heavily impacted area by 2019-nCoV, received 50,340 COVID-19 patients and 3869 patients with critical clinical syndrome died^4^. According to the statistics of the National Health Commission of China, up to April 27th, 2020, Wuhan has cleared all severe 2019-nCoV cases. With this exhilarating news, we need to understand the importance of critical patients by estimating the correct clinical condition development and establishing rational treatment plans.

Based on the “Preventing forward” idea proposed by life-threatening specialists in Wuhan, the critical COVID-19 death risk estimation model was built. Many scholars found that changes in blood, high-sensitivity C-reactive protein (hs-CRP), and d-dimer, combined with a patient’s age and medical complications, had distinct instructive value for critical COVID-19 patient prognosis^5–7^. There is an urgent need to identify clinical and laboratory predictors of the progression to fatal forms of the disease. These predictors will enable risk stratification, guide interventional studies, target patients at an increased risk of developing severe disease, and optimize the allocation of limited human and technical resources in an ongoing pandemic. However, the prediction model on the prognosis of critical COVID-19 patients still requires further study. The purpose of this study is to explore the influencing factors on critical COVID-19 patients’ prognosis and construct a nomogram model to predict the mortality risk.

## Results

### Patient characteristics

A total of 102 COVID-19 critical patients were included in the study and divided into the survival group (50) and death group (52). There were 63 males and 39 females, 49 cases with hypertension, 21 cases with diabetes, 16 cases with coronary heart disease, and 9 cases with smoking habits. The demographic and clinical characteristics were listed in Table 1. Age, sex, coronary heart disease history, body temperature, peripheral white blood cell count (WBC), lymphocyte count (L), platelet count (PLT), hs-CRP, estimated glomerular filtration rate (eGFR), d-dimer (D-D), and troponin I (TnI) were significantly different between the two groups (*p* < 0.05).

**Table 1:** Comparability of clinical data and laboratory indicators between the death and survival group.

Items	Survival group (n = 50)	Death group (n = 52)	*t/Z/χ*^*2*^	*p*
Age (years)	65.00(14.50)	74.50(12.75)	− 4.68	< 0.001
Sex (M/F)	24/26	39/13	7.87	0.005
Hypertension (Y/N)	20/30	29/23	2.54	0.111
Diabetes (Y/N)	11/39	10/42	0.12	0.730
Coronary heart disease (Y/N)	3/47	13/39	6.96	0.008
Smoking habit (Y/N)	3/47	6/46	0.41	0.524
Body temperature (℃)	38 (1.63)	37 (1.50)	− 2.49	0.013
WBC (*10^9^/L)	5.68 (2.83)	8.06 (7.22)	− 4.55	< 0.001
L (*10^9^/L)	1.26 (0.49)	0.75 (0.39)	5.83	< 0.001
PLT (*10^9^/L)	240 (129.75)	144.50 (121.25)	− 4.90	< 0.001
Hs-CRP (mg/L)	18.4 (59.90)	113.30 (93.20)	− 6.51	< 0.001
eGFR (ml/min)	92.55 (15.30)	66.20 (38.05)	− 4.17	< 0.001
d-Dimer (μg/mL)	0.80 (1.43)	19.09 (18.97)	− 6.35	< 0.001
TnI (μg/L)	2.55 (4.73)	40.75 (652.83)	− 6.77	< 0.001

Continuous variables with normal distribution were expressed as the mean ± standard deviation (SD), non-normal variables were expressed as the median (interquartile range (IQR)), and categorical data were expressed as number and percentage. The independent sample Student's t-test was used to compare the means of two continuous normally distributed variables. The means of two non-normally distributed variables were compared with the Mann–Whitney U test. The frequencies of categorical variables were compared by the *χ*^*2*^ test.

### Logistic regression analysis of critical COVID-19 patient mortality risk

As indicated in Table 2, the personal characteristics logistic regression analysis (Model 1) of age, sex, smoking habits, and body temperature concluded that age, sex, and body temperature were risk factors affecting critical COVID-19 patient mortality. The complication logistic regression analysis (Model 2) of hypertension, diabetes, and coronary heart disease concluded that coronary heart disease was a risk factor affecting critical COVID-19 patient mortality. The clinical indicators logistic regression analysis (Model 3) of peripheral WBC, L, PLT, hs-CRP, eGFR, D-D, and TnI concluded that WBC, L, PLT, hs-CRP, and eGFR were risk factors affecting critical COVID-19 patient mortality. The integration indicators logistic regression analysis (Model 4) of age, sex, smoking habits, body temperature, hypertension, diabetes, coronary heart disease, WBC, L, PLT, hs-CRP, eGFR, D-D, and TnI, concluded that age, WBC, L, PLT, and hs-CRP were factors affecting critical COVID-19 patient mortality risk.

**Table 2:** Multivariate logistic regression analysis for risk of death in critical COVID-19 patients.

Items	OR (95%CI)	*p*
**Model 1**		
Age	1.106 (1.048–1.168)	< 0.001
Sex	3.885 (1.409–10.717)	0.009
Body temperature	0.513 (0.317–0.832)	0.007
**Model 2**		
Coronary heart disease	5.222 (1.388–19.651)	0.015
**Model 3**		
WBC	1.326 (1.032–1.702)	0.027
L	0.064 (0.007–0.598)	0.016
PLT	0.989 (0.978–1.000)	0.041
hs-CRP	1.030 (1.010–1.051)	0.003
eGFR	0.953 (0.919–0.987)	0.008
**Model 4**		
Age	1.135 (1.045–1.232)	0.003
WBC	1.313 (1.027–1.678)	0.030
L	0.048 (0.005–0.485)	0.010
PLT	0.986 (0.974–0.998)	0.023
hs-CRP	1.028 (1.008–1.049)	0.006

Model 1: logistic regression analysis of age, sex, smoking habits, body temperature; Model 2: logistic regression analysis of the presence of hypertension, diabetes, and coronary heart disease; Model 3: logistic regression analysis of peripheral white blood cell count (WBC), lymphocyte count (L), platelet count (PLT), high-sensitivity C-reactive protein (hs-CRP), estimated glomerular filtration rate (eGFR), d-dimer (D-D), and troponin I (TnI); Model 4: logistic regression analysis of all items above.

### Nomogram construction and validation

**Figure 1:**
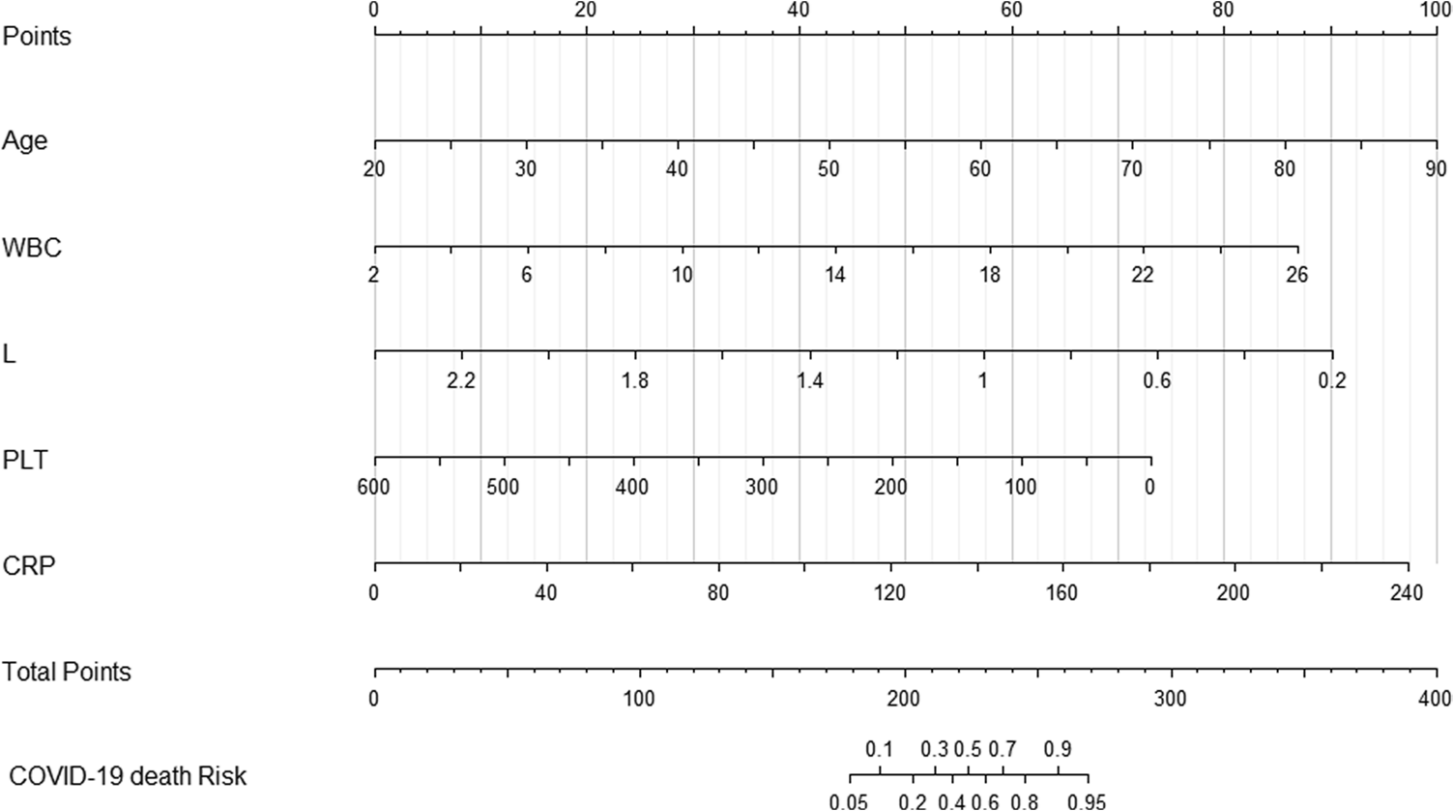
Individualized predictive nomogram model in predicting the risk of death in critical COVID-19 patients. The figure was created using R software version 3.6.1 (http://www.R-project.org).

Critical COVID-19 patient mortality risk factors concluded from Model 4 were input into the software R. An estimation nomogram of critical COVID-19 patient mortality risk was then built. Each item in the model corresponded to a score, the sum of each item score became a total score. The point on the critical COVID-19 patient mortality risk axis corresponds to patient mortality risk. In the nomogram, the total scores ranged from 179 to 270, with corresponding mortality risks of 0.05 to 0.95. The higher the score, the higher the mortality risk (Fig. 1). In addition, the area under the receiver operating characteristic (ROC) curve in Table 3 was utilized to calculate the accuracy for different test indexes on the prediction of survival. The results showed that the area under the curve of the prediction model was the largest. Across all test indexes, hs-CRP had the largest area under the curve, which was the closest to the prediction model.

**Table 3:** ROC curves of critical COVID-19 patients.

Items	AUC (95%CI)	*p*
Age	0.764 (0.669–0.859)	< 0.001
Body temperature	0.769 (0.675–0.863)	0.018
L	0.209 (0.118–0.300)	< 0.001
PLT	0.216 (0.125–0.306)	< 0.001
Hs-CRP	0.879 (0.815–0.944)	< 0.001
Prediction model	0.958 (0.923–0.993)	< 0.001

**Figure 2:**
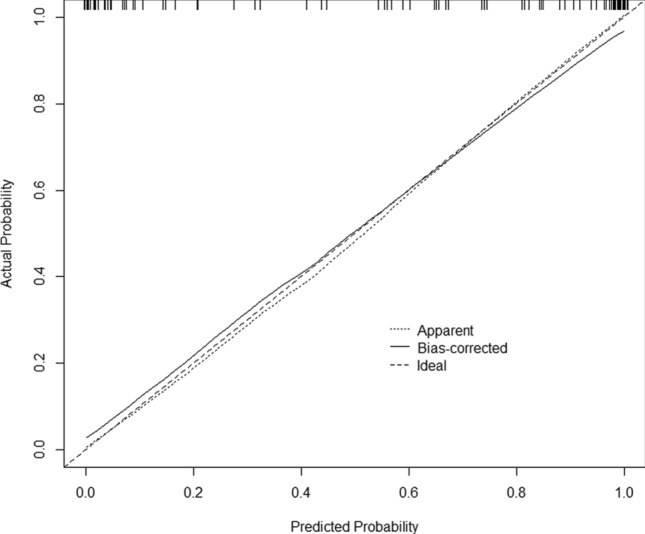
Calibration plot of nomogram model in predicting risk of death in critical COVID-19 patients. The figure was created out using R software version 3.6.1 (http://www.R-project.org).

The C-index for this nomogram was 0.958 (95% CI 0.923–0.993), indicating a good discriminative ability. The calibration curve (Fig. 2) showed consistency between prediction probability and actual probability, which indicates the accuracy of the nomogram in estimating critical COVID-19 mortality risk.

## Discussion

The new coronavirus disease, COVID-19, is an acute respiratory infectious disease caused by the SARS-CoV-2 virus. As of January 22nd, 2021, there have been more than 9.8 million confirmed cases of COVID-19 globally, and the number of deaths had reached more than 2 million^8^. Investigations have found that only 80% of confirmed cases showed mild illness, but an admission survey of 166 British hospitals showed that around 33% of diagnosed patients required hospitalization and 45% of patients in intensive care died from the disease^9^.

Based on the comparison of various indicators of COVID-19 in patients in the survival group, our findings indicated that patients with advanced age, high WBC, low L, low PLT, and high hs-CRP were at high risk of mortality. A nomogram model including age, WBC, L, PLT, and CRP was constructed, which may be used for early and intuitive identification and evaluation of the prognosis of critical COVID-19 patients and provide a basis for personalized prevention and treatment.

COVID-19 infections appear in clusters, and are more likely to affect elderly males with comorbidities^10–12^. We analyzed patient's comorbidity and Model 2 showed that coronary heart disease was a risk factor for death in patients with severe COVID-19 (OR = 5.222). Zheng et al*.*^13^ found that patients with cardiovascular disease have increased angiotensin converting enzyme 2 (ACE-2), compared to healthy individuals, while Goldstein et al*.*^14^ have discussed that angiotensin receptor blocker’s (ARB’s) facilitate viral entry by increasing the expression of ACE-2 receptors resulting in greater disease severity. The virus causing COVID-19 is SARS-CoV-2, which is a new type of coronavirus which infects cells with ACE-2 as the receptor^15^. Changes in ACE-2 levels in the body can affect the pathogenicity of SARS-CoV-2. The high expression of ACE-2 enhances the ability of SARS-CoV-2 to infect the body, and the level of ACE-2 in patients with coronary heart disease is significantly higher than patients without. Similarly, the large percentage of patients with hypertension undertaking angiotensin-converting enzyme inhibitors (ACEIs) or ARBs is a remarkable characteristic among those presenting severe COVID-19 manifestations. However, Cao et al*.* have shown that the East Asian population has a higher expression than other countries^16,17^. Only 16 of the 102 patients in this study had coronary heart disease, therefore the results of the survey were relatively limited. Whether coronary heart disease is associated with increased mortality in COVID-19 requires further study and there are also contradictory scenarios suggesting that the use of ACEIs/ARBs may exacerbate the deleterious conditions of the infection, therefore, this remains an area requiring further investigation.

Many studies have considered high temperature to be related to the progression of COVID-19^13,14,18^. In this study, we found that body temperature in the death group was slightly lower than in the survival group. During the outbreak of COVID-19 in Wuhan, many patients were not able to be admitted into hospital. Many underwent cooling and other treatments at home at that time, so the body temperature measured after patients were admitted in this study may have been affected. As with the study by Chen et al*.,* patients were also from Wuhan city, meaning there is a small number of fever patients included^19^_._ Model 1 showed that body temperature was different between the two groups in this study (OR = 0.513), but Model 4 did not find a difference in body temperature after adjusting for more factors, suggesting that the association between body temperature and prognosis may not be clear and requires further study.

Nomograms can show the probability of clinical events in statistical prediction models as simple scores through graphics, which can be used to predict prognosis by estimating clinical events and integrating significant prognostic factors in numerous diseases^20–22^. In the present study, we constructed a nomogram including age, WBC, L, PLT, and hs-CRP, which were screened through multivariate logistic regression analysis (Model 4). Individuals with higher total score from the nomogram had a greater risk of an unexpected prognosis. Through discriminatory capacity and calibration ability analysis, the results showed that this model was stable and had a high ability to predict prognosis of critical COVID-19 patients (C-index: 0.958, 95% CI 0.923–0.993). The calibration curve (Fig. 2) showed that the predicted probability and the actual probability were consistent with good accuracy and differentiation, indicating that the nomogram model can accurately predict the risk of death of critical COVID-19 patients.

Compared to other studies which also constructed a nomogram for assessing survival in COVID-19 patients^19,23,24^, the indicators used in the current study are more basic and easier to obtain. However, there remains limitations. First, this was a retrospective study with a relatively limited number of patients, the causal relationship between the influencing factors and critical COVID-19 patients’ prognosis was not certain. Second, although the confounding factors were adjusted by logistic regression, and the nomogram model was demonstrated to exhibit appropriate discriminatory capacity and calibration ability, the lack of symptomatic data and specific medications may affect the conclusions to some extent. Third, the participants were recruited from a single hospital, we therefore conducted an internal validation but lack externally validation.

## Materials and methods

### Sample collection

This was a retrospective study with the samples being obtained from Tongji Hospital, Guanggu District, Wuhan City, Hubei, China from February 2020 to March 2020. A total of 102 critical COVID-19 patients were included and divided into the survival group and death group according to patient prognosis. The diagnosis and clinical classification of COVID-19 were based on the guidelines of the treatment of new coronavirus pneumonia (version 7) published by the National Health Commission of China^25^. The diagnosis was established on the basis of (1) epidemiological history, (2) fever and/or other respiratory symptoms, (3) presence or absence of imaging findings of novel coronavirus pneumonia, and (4) real time fluorescence RT-PCR for SARS-CoV-2 nucleic acid which yielded positive results. As for clinical classification, patients were deemed critical if they met at least one of the following criteria: (1) shortness of breath, with respiratory rate ≥ 30 breaths/min; (2) oxygen saturation (at resting state) ≤ 93%; (3) PaO_2_/FiO_2_ ≤ 300 mmHg; (4) radiographical imaging showing lesion progression more than 50% within 24–48 h; or (5) respiratory failure, shock, or other organ failures. In addition, all critical patients were hospitalized for ≥ 24 h. This study was approved by the Institutional Ethics Committee of Union Hospital, Tongji Medical College, Huazhong University of Science and Technology (TJ-IRB20201014); the requirement for informed consent was exempted by the Ethics Committee. All methods were carried out in accordance with relevant guidelines and regulations.

### Data collection and measurement

Demographic data including age, sex, smoking habits, body temperature, and comorbidity data including hypertension, diabetes, and coronary heart disease were collected. Fasting blood samples were extracted by venipuncture to collect clinical indicators in a core laboratory with a standard protocol. An ADVIA2400L automated biochemistry analyzer (Siemens AG, Munich, Germany) was used to measure peripheral WBC, L, PLT, hs-CRP, and D-D. A VITROS5600 (Johnson & Johnson, American) biochemistry analyzer was used to measure TnI. Clinical indicators were measured in a core laboratory with a standard protocol.

### Statistical analysis

All statistical analyses were carried out using PASW Statistics 19.0 (SPSS, Inc., Somers, NY, USA) and R software version 3.6.1 (http://www.R-project.org). The Kolmogorov–Smirnov test was used to measure data normality. If the data satisfied normal distribution, mean ± standard deviation (SD) was shown and a T-test was used to find differences between two groups. Abnormally distributed data was shown as median (IQR), while a risk-sum test being used for comparison (Mann–Whitney U test). Categorical data were expressed as number and percentage and compared with the Chi-square test. Furthermore, logistic regression analysis by the stepwise method (Forward wald) was used. A nomogram was constructed based on the results of the logistic regression to predict the mortality risk using the nomogram function in the R software. Harrell’s C-statistic was used to calculate C-index and to verify nomogram discrimination. Calibration curves were used to verify nomogram conformity. C-index and calibration curve were obtained by Bootstraps (1,000 multiple sampling). A two-sided *p* < 0.05 was considered statistically significant.

## Data Availability

The data that support the findings of this study are available from the corresponding author upon reasonable request.
